# Knowledge, attitude and practice towards exclusive breastfeeding among lactating mothers in Mizan Aman town, Southwestern Ethiopia: descriptive cross-sectional study

**DOI:** 10.1186/s13006-016-0062-0

**Published:** 2016-02-27

**Authors:** Niguse Tadele, Frehiwot Habta, Dinu Akmel, Eyerus Deges

**Affiliations:** Mizan Tepi University, College of Medicine and Health Sciences, Department of Nursing, Mizan Aman town, Ethiopia

**Keywords:** Exclusive breastfeeding, Knowledge, Attitude, Practice, Ethiopia

## Abstract

**Background:**

Exclusive breastfeeding (EBF) is the best nutrition for children during the first six months of life. However EBF remains a challenge. The aim of the study was to assess Knowledge, Attitude and Practice towards EBF among breastfeeding mothers in Mizan Aman town, South West Ethiopia.

**Methods:**

A community based cross-sectional study was conducted among 350 mothers in Mizan Aman town, South West Ethiopia, in April 2015 using a structured interviewer-administered questionnaire using ‘recall since birth’ method. Systematic random sampling was used to select the study participants and descriptive statistics were conducted.

**Results:**

Three hundred and fourteen breastfeeding mothers with their index child less than 2 years were enrolled. Even though 93.6 % of study participants had heard about EBF, only 34.7 % were knowledgeable about the recommended duration. About 89.5 % had a positive attitude, but only 59.3 % believed that only EBF is enough for child up to six months and 26.4 % of children were exclusively breastfed for six months.

**Conclusion:**

The majority of mothers knew about EBF and had a positive attitude towards EBF but did not know the recommended duration or that EBF is sufficient for six months. We suggest improving access to information about recommended infant feeding guidelines and fulfilling the minimum enabling conditions.

## Background

Exclusive breastfeeding (EBF) is defined as “an infant’s consumption of human milk with no supplementation of any type (no water, no juice, no nonhuman milk, and no foods) except for vitamins, minerals, and medications until six months” [1]. EBF for six months is important for both infant and maternal health. Infants who are not exclusively breastfeeding are more likely to develop gastrointestinal infections, not only in developing but also in industrialized countries. The risk of mortality due to diarrhea and other infections can increase many-fold in infants who are either partially breastfed or not breastfed at all [1]. During the first two months of life, infants who are not breastfed are nearly six times more likely to die from infectious diseases than infants who are breastfed; between 2 and 3 months, non-breastfed infants are 4 times more likely to die compared to breastfed infants [2, 3].

Infant and young child feeding practices directly affect the nutritional status of children under two years of age and, ultimately, impact child survival. Worldwide, more than nine million children under five years of age die each year [4]. One in every 17 Ethiopian children die before the first birthday and one in every 11 children dies before their fifth birthday [5].

Over two-thirds of these deaths are associated with inappropriate feeding practices and occur in the first year of life. Optimal breastfeeding (early and exclusive breastfeeding) has the potential to prevent over 800,000 deaths (13 % of all deaths) in children under five in the developing world. Whereas, complementary feeding interventions alone were estimated to prevent almost one fifth of deaths in children aged years [4, 5].

Rates of EBF are suboptimal in many countries. In Saudi Arabia only by 8.3 % (*n* = 32) of the 384 participants EBF for 6 months [6]. In a study in Congo, 87.5 % of infants were EBF during the maternity stay, but by six months, only 2.8 % (*n* = 12) of infants were exclusively breastfed [7]. In a study done in Nigeria, the majority (88.0 %) of the respondents had heard about EBF and hospital was the source of information, and more than 50 % of the women had a positive attitude towards breastfeeding [8].

### Ethiopian context

The duration of breastfeeding in Ethiopia is long, but EBF during the first six months after birth is not widely practiced. Currently, mothers exclusively breastfeed approximately half of children less than six months (52 %). Among sub-groups the percentage of young children who are exclusively breastfed decreases sharply from 70 % of infants age 0–1 month to 55 % of those aged 2–3 months, and further, to 32 % among infants 4–5 months. In addition to breast milk, 19 percent of infants less than six months are given plain water only, while 14 percent receive milk in addition to breast milk, and four percent are given non-milk liquids and juice [5].

A study in Addis Ababa, Ethiopia found the prevalence of EBF under six months was 29.3 % [9]. A study in Arbaminch, Ethiopia showed that 55.6 % exclusively breastfed their children for six months. Three-hundred forty one (89 %) mothers gave colostrum, however a small number of mothers considered colostrum to be expired breast milk and discarded it [10]. A study in Bedele, Ethiopia found that the majority of mothers, 91.8 %, knew the importance of EBF, and 87.3 % of mothers had a positive attitude and strongly agreed that the EBF is advantageous for infants aged less than six months. Only 43.6 % of mothers practiced EBF for the first six months after delivery [11]. A study in Southern Ethiopia found that 56.7 % of mothers living with HIV (Human Immunodeficiency Virus) had a favorable attitude towards EBF and nearly half (48.2 %) of mothers exclusively breastfeed their infants [12]. A study in Harar, Ethiopia indicated EBF was 51.8 % [13]. A study in Gondar showed EBF rate of 35.9 %. Among the respondents, nearly half (49.4 %) exclusively breastfed for only 3 months or less [14]. In a study done in Debre Birhan Ethiopia 68.6 % of mothers practiced EBF to six months, 83.4 % of mothers were knowledgeable of recommended duration of EBF and about 97.5 % of mothers had a positive attitude towards EBF practice [15]. In a study done in Ambo Ethiopia, the prevalence of EBF was 82.2 %, and 90.8 % of mothers were knowledgeable about EBF [16].

The aim of the study was to assess Knowledge, Attitude and Practice (KAP) towards EBF among lactating mothers in Mizan Aman Town, South West Ethiopia, who had a child less than two years during April 2015. The findings will help to describe EBF practice and reveal areas for further study and will assist health care professionals, health extension workers and community health promoters to understand EBF practice in the study area and help them to prioritize and focus their efforts. Additionally, since there is a limited research in the study area, this study can be used as a benchmark for further studies.

## Methods

This community based cross-sectional descriptive study using ‘recall since birth’ method was conducted in April 2015, to assess Knowledge, Attitude and Practice towards exclusive breastfeeding among lactating mothers in Mizan Aman Town,South West Ethiopia. Mizan Aman town is located in Southern Nations Nationalities and Peoples Regional (SNNPR) State, 565 kilometers from Addis Ababa, the capital city of Ethiopia at a latitude and longitude of 7° N 35° E and an elevation of 1451 meters. Administratively, the town is structured into five kebeles (smallest administrative units) and two sub-cities. The economy of the town is widely based on trade of cash crops especially of coffee. There is 24 hours electricity, access to potable water, digital telephone access, postal service and a 70 bed hospital, health center and different private clinics, pharmacies and drug stores. It is also a location of three higher education institutions; Aman Health Sciences College, Aman Polytechnic College and Mizan Tepi University. The population of Mizan Aman town was estimated to be 47,776 in 2015. The number of children under 2 years of age is estimated to be 2475 (about 5 % of the total population) [17].

Firstly, population census was conducted in all five kebeles (three urban and two rural) and identified 1800 households of mother with index infant aged less than 2 years and currently breastfeeding their infants. A sampling frame was prepared and study subjects were selected from every fifth household using systematic random sampling techniques. During data collection,36 mothers and their index infants were replaced by the immediate next household due to incomplete or non- responses. Data were collected from 314 mothers with their index infant aged less than 2 years after explaining the purpose of the study, informed consent was obtained from each respondent.

The sample size was calculated using a single population proportion formula [n = [[Z [1 − *α*/2]]^2^ X pX [1 − p]]/d^2^] with the following assumptions: 52 % prevalence of EBF at national level [5], 95 % confidence level, 5 % degree of desired precision, finite population correction factor formula [*n* = no/1 + no/N] since the total number of breastfeeding mothers in the city [N] was 2475 and 5 % for non-response rate. A total of 350 mothers were selected by systematic random sampling from households who had a child of less than two years old.

A structured interviewer administered questionnaire adopted from the EDHS (Ethiopian Demographic and Health Survey) and other literature was used to collect data. Three graduating class Bsc nursing students collected the data. The interviews were conducted in the compound of the mother’s home. The collected data were checked for completeness, coded and entered in to a computer. Statistical analysis was carried out using SPSS for Windows version 20.0. The data were summarized by descriptive statistics using the frequency, percentage and tables for categorical variables.

### Ethical considerations

Ethical clearance was obtained from Mizan Tepi University, College of Health Sciences, Support letter was written by Mizan Tepi University to Mizan Aman City administration and official permission was obtained.

## Results

### Sociodemographic characteristics

Three hundred and fourteen mother-child pairs participated in the study with a response rate of 89.7 %. The majority (68.5 %) were between 20–30 years, were married (80.6 %), had three or more children (58.2 %) and were followers of Orthodox Christianity (53.8 %). About one in five could not read (22.3 %) and 31.5 % earned less than 1 USD per day (Table 1).

**Table 1:** Sociodemographic characteristics of study participants Mizan Aman, Ethiopia, April 2015 (*n* = 314)

Characteristics		Frequency	Percentage
Maternal age (years)	<20	75	23.9
	21–25	92	29.3
	26–30	123	39.2
	>31	24	7.6
Maternal marital status	Married	253	80.6
	Single	24	7.6
	Divorced	22	7.0
	Widowed	15	4.8
Maternal religion	Orthodox	169	53.8
	Protestant	74	23.6
	Muslim	53	16.9
	Other Christian^a^	18	5.7
Maternal ethnicity	Bench	105	33.4
	Kaffa	90	28.7
	Amhara	69	22.0
	Other^b^	50	15.9
Maternal occupation	Housewife	97	30.9
	Gov’t employee	88	28.0
	Daily laborer	74	23.6
	Merchant	48	15.3
	Student	7	2.2
Maternal education	Can’t read	70	22.3
	Read and write	75	23.9
	1–8 years	72	22.9
	9–12 years	62	19.8
	University/College	35	11.1
Average monthly income	<30$ (600 ETB)	99	31.53
	> = 30$	215	68.47
Number of children	<3	131	41.8
	> = 3	183	58.2

^a^Catholic, Jovha and Adventist

^b^Silte, Gurage, Tigre, Hadya, Wolayita, Sheka, Yem, Kembata, Dizi, Surma

### Knowledge of respondents about EBF

The majority 294 of study participants had received information about EBF (93.6 %) and their main source of information were health professionals (62.7 %). Concerning initiation, the majority replied that breast milk should be started immediately after birth (73.2 %). The majority of mothers replied frequent sucking is helpful for milk production (52.2 %), although one in five mothers (20.1 %) had no idea about the relationship between sucking and milk production.

Regarding the duration of EBF, only about one third of mothers interviewed (34.7 %) mentioned up to six months. About half of mothers said there is adequate breast milk if the child is satisfied (53.8 %). One quarter of mothers knew that EBF for six months protects their child from diarrhea (27.3 %); 32 % of mothers responded that EBF can be used as a contraceptive, while 16.7 % didn’t think that it could be used as a contraceptive (Table 2).

**Table 2:** Knowledge of study participants towards exclusive breastfeeding, Mizan Aman, Ethiopia, April 2015 (*n* = 314)

Variable		Frequency	Percent
Ever heard about EBF?	Yes	294	93.6
	No	20	6.4
Source of information about EBF?	Health institution	197	62.7
	Friends	29	9.3
	Mass media	63	20
	Other^a^	25	8
When should breastfeeding after delivery started?	Immediately	230	73.2
	2–24 hours	75	23.9
	After 24 hours	9	2.9
Does frequent sucking help for milk production?	Yes	164	52.2
	No	87	27.7
	No idea	63	20.1
For how long is EBF needed?	<6 month	164	52.2
	About 6 months	109	34.7
	Beyond 6 months	41	13.1
How did you know when there is adequate breast milk?	If the baby is satisfied	169	53.8
	If the baby slept after feeding	89	28.4
	Others^b^	56	17.8
Does EBF for 6 month prevent child from diarrhea?	Yes	86	27.3
	No	193	61.3
	Don’t know	35	11.4
Does EBF prevent pregnancy?	Yes	100	32
	No	53	16.7
	Don’t know	161	51.3

^a^From school, family, neighborhoods

^b^If the mother eats well, is healthy and takes enough rest it will be enough

### Attitude of respondents towards EBF

We found that more mothers (*n* = 281; 89.5 %) said they preferred to feed their children only breast milk than the number who were aware of the recommendation to exclusively breastfed for six months (*n* = 109; 34.7 %). Among those mothers who said they preferred to feed their children only breast milk, 73.0 % (*n* = 205) said that EBF is better than artificial feeds.

Most mothers (59.3 %) agreed that only EBF is enough up to 6 months of age. Also a high proportion, 60.2 % (*n* = 189) of mothers believed colostrum should not be discarded. The majority of mothers, 59.6 % (*n* = 187) did not feel comfortable when they gave extra foods other than the breast, and about half the mothers (*n* = 182; 58.0 %) agreed that exclusively breastfed children are healthier than non-exclusively breastfed children (Table 3).

**Table 3:** Attitude of study participants towards exclusive breastfeeding, Mizan Aman, Ethiopia, April 2015 (*n* = 314)

Variables		Frequency	Percent
What do you prefer to feed your baby for the first 6 months?	Breast milk only	281	89.5
	Breast and other food items	33	10.5
Do you think that EBF is better than artificial feeding (*n* = 281)?	Yes	205	73.0
	No	67	23.8
	Don’t know	9	3.2
Do you believe that the first milk [colostrums] should be discarded?	Yes	125	39.8
	No	189	60.2
Do you agree that only EBF is enough for child up to 6 months?	Agree	186	59.3
	Disagree	128	40.7
How did you feel when you give extra food other than breast to your child?	Didn’t feel comfort	187	59.6
	Comfortable with it	127	40.6
Why you are not comfortable with extra feeding other than breast (*n* = 187)?	Not sufficient to meet child’s demand	82	43.9
	It’s not necessary for child	75	40.1
	Complain feeling of pain	30	16.0
Do you agree that child less than 6 month who is exclusively breastfed is healthier than child who takes additional food?	Yes	182	58.0
	No	79	25.1
	I don’t know	53	16.9

### Exclusive breastfeeding practices among respondents

In this study, all mothers reported breastfeeding their child. The majority of participants (*n* = 188; 59.9 %) had initiated breastfeeding immediately, while 4.5 % (*n* = 14) initiated breastfeeding one day after giving birth. Two thirds of mothers (*n* = 209; 66.6 %) were breastfeeding on demand and the majority had not given any prelacteal feeds to their newborn baby (*n* = 243; 77.4 %). Of those who gave prelacteal feeds, about half gave plain water (*n* = 34; 47.8 %). Exclusive breastfeeding was reported only by 26.4 % (*n* = 83) of mothers, while 50.2 % (*n* = 116) gave additional cow milk to their infant prior to six months of age (Table 4).

**Table 4:** Exclusive breastfeeding practices of study participants, Mizan Aman, Ethiopia, April 2015 (*n* = 314)

Variables		Frequency	Percent
Have you breastfeed your last child?	Yes	314	100.0
When did you start breastfeeding after delivering your last child?	Immediately	188	59.9
	Between 2 and 24 hours	112	35.7
	After 24 hour	14	4.5
How frequently did you breastfed your last child?	On demand	209	66.6
	Regularly	101	32.2
	Randomly	4	1.3
Have you given your last baby anything before initiating breastfeeding?	No	243	77.4
	Yes	71	22.6
What was given to your last baby before breast milk after delivery?	Plain water	34	47.9
	Cow milk	25	35.2
	Butter	12	16.9
What was given to your last child starting from birth to 6 month?	Cow and breast milk	116	36.9
	Breast milk only	83	26.4
	Formula	23	7.3
	Other^a^	92	29.3

^a^Juice, tea, soup & plain water

## Discussion

This study investigated KAP of EBF among mothers with children less than two years of age. The majority of study participants had heard about EBF (93.6 %) which was similar to other studies conducted locally and in other countries. A study in Bedele, Ethiopia found that the majority of mothers (91.8 %) knew the importance of EBF [11]. A study in Ambo Ethiopia found 90.8 % of mothers were knowledgeable about EBF [16] and a Nigerian study showed the majority (88.0 %) of the respondents had heard about EBF [8]. Although the majority of respondents had good knowledge about EBF, only about one third of mothers (34.7 %) mentioned the recommended duration of EBF, which was lower than a study in Debre Birhan Ethiopia in which 83.4 % of mothers were knowledgeable about the recommended duration of EBF [15].

In this study even though only 59.3 % believed that EBF is enough up to 6 months, the majority of mothers (89.5 %) preferred to feed their child breast milk only, which may be attributable to the recommendations and enforcements in some cases by health extension workers, the Health Development Army and other health professionals. Additionally most of the interviewed mothers could not afford to purchase extra food. This finding was similar to a study in Bedele, Ethiopia which found that the majority of mothers (87.3 %) had a positive attitude and strongly agreed that EBF is advantageous for infants aged less than six months [11], and a study in Debre Birhan Ethiopia which found 97.5 % of mothers had a positive attitude towards EBF [15]. The findings of this study showed a higher number of mothers with a favorable attitude towards EBF than in Southern Ethiopia (56.7 % of mothers had a favorable attitude towards EBF) [12] and in Nigeria (50 % of women had a positive attitude towards breastfeeding) [8]. This difference might be due to differences in sociodemographic characteristics of study samples such as differences in ethnic background, economic status, type of employment and educational status and differences in study period and study area.

In this study EBF was reported only by 26.4 % of mothers, which is lower than the national report in which 52 % of children less than six months were exclusively breastfed. This difference might be attributable to differences in sample: in the national study most of the surveyed areas were rural (437 rural areas from the total of 624 enumeration areas) while this study included urban kebeles and rural kebeles which are closer to town, and the national study included samples from all regions of Ethiopia including individuals with different sociocultural backgrounds while the present study was conducted in a single city administration [5].

EBF findings were lower than studies in other parts of Ethiopia including Arbaminch, Bedele, Southern Ethiopia, Harar, Gondar, Debre Birhan and Ambo which showed that 55.6, 43.6, 48.2, 51.8, 35.9, 68.6 and 82.2 % of mothers exclusively breastfed their children for 6 months respectively [10–16]. This difference might be due to lack of knowledge about optimal breastfeeding practices in which optimal breastfeeding was reported only about 34.7 %, due to field and home activities and due to the resumption of income-generating activities, might be due to difference in sociodemographic characteristics, differences in cultural habit and differences in the study area and period. This difference might also probably due to the chance to afford replacement feeding, so as to avoid breastfeeding. Additionally methodological differences occur because some studies like the DHS [5] calculated exclusive breastfeeding over a 24 hour recall period which appears to exaggerate the prevalence of EBF at six months [18], whereas this study used recall since birth method of defining EBF.

Our EBF findings were similar to a study in Addis Ababa, Ethiopia which found 29.3 % prevalence of EBF under six months [9], while it was higher than a study in Abha female educational district, Saudi Arabia in which EBF for 6 months was reported only by 8.3 % of participants (*n* = 32) [6] and a study in Kinshasa, Congo that found only 2.8 % of infants were exclusively breastfed (*n* =12) [7].

### Limitations

This study shares the limitation of a cross-sectional study design. Additionally this study may introduce social desirability and recall bias since infants up to 2 years were included. This study was also not supplemented with any qualitative data.

## Conclusions

In this study even though the majority of mothers were knowledgeable about EBF and had a positive attitude towards EBF, knowledge with recommended duration of EBF, attitude of mothers towards sufficiency of EBF and EBF for 6 months was low. This study concluded poor knowledge and practice to EBF for the first six months postpartum among women in Mizan Aman Town.

Based on the findings of this study, we have the following recommendations:

- The fact that a large proportion of mothers practiced suboptimal feeding practices 11 years after the development of the national Infant and young child feeding (IYCF) guideline [19] indicates the need for strengthening the behavior change communication on optimal IYCF practices;
- Attention in health planning should be given to EBF promotion: health care providers and decision-makers should comprehensively address issues to improve EBF practices in the community.
- Improving access to information on recommended infant feeding during routine maternal and child health services and strengthening the nutrition counseling during antenatal and postnatal sessions.
- Educating mothers about optimal child feeding practices at different occasion like holy day and other gatherings is better opportunity to enhance mothers’ knowledge of child feeding practices.
- In order to promote optimal duration for EBF, the WHO advocated for minimum enabling conditions such as paid maternity leave, part-time work arrangements, facilities for expressing and storing breast milk and breastfeeding breaks for women in paid employment [20] is recommended.

## Abbreviations

EBF: exclusive breastfeeding
EDHS: Ethiopian Demographic and Health Survey
HHs: households
HIV: human immunodeficiency virus
IYCF: infant and young child feeding
KAP: knowledge, attitude and practice
SNNPR: Southern Nations Nationalities and Peoples Region
USD: United States Dollar
WHO: World Health Organization
